# Supplementary material for: Natural Bred ε2-Phages Have an Improved Host Range and Virulence against Uropathogenic Escherichia coli over Their Ancestor Phages
Source: Antibiotics (Basel). 2021 Nov 1;10(11):1337. doi: 10.3390/antibiotics10111337 (PMC8614997; doi:10.3390/antibiotics10111337)
Supplement: Supplementary file 1 [file antibiotics-10-01337-s001.zip › Table S1.pdf]

Table S1. Composition of the 79 *E. coli* strains originating from urinary tract infections used for initial HR determination of wastewater isolated phages

serotype	number
O25:H4	37
O1:H25	1
O100:H4	2
O101:H9	1
O15:H18	1
O153:X	1
O18:H1	1
O18:H7	1
O2:H6	3
O20.H9	1
O23.X	1
O24.H4	1
O25.H1	2
O39.X	1
O4.H5	1
O6.H1	1
O6.H18	2
O6.X	2
O73.H18	1
O75.H7	1
O8.H10	1
O8.H7	1
O9.H19	1
O9.H25	1
O9.H30	1
O9.H4	1
O9.H9	1
X.H14	1
X.H18	1
X.H19	1
X.H4	1
X.H5	1
X.H6	1
unknown	4
