# Supplementary material for: Natural Bred ε2-Phages Have an Improved Host Range and Virulence against Uropathogenic Escherichia coli over Their Ancestor Phages
Source: Antibiotics (Basel). 2021 Nov 1;10(11):1337. doi: 10.3390/antibiotics10111337 (PMC8614997; doi:10.3390/antibiotics10111337)
Supplement: Supplementary file 1 [file antibiotics-10-01337-s001.zip › Table S2.pdf]

Table S2. Overview of the 47 *E. coli* strains used for comparison of PHR and KHR and further characterization

Strain	Phylogenetic group	Sequence type	Antibiotic resistance gene	antibiotic (co-) resistance
Af23[#]	A	10	*mcr-1*, *bla*$_{TEM-1}$	AMX, CIP, CS, SXT, TET
K12[#]*		10		
CHD28		90	*bla*$_{CTX-M-15}$, *bla*$_{OXA-1}$	AMP, CTX, GEN
CHD1[#]		93	*bla*$_{TEM-1B}$, *bla*$_{CTX-M-1}$	AMP, CTX
CDF8		167	*mcr-1*, *bla*$_{CTX-M}$	AMP, AMX, CIP, CS, CTX, KAN, NAL
Af49[#]		226	*mcr-1*, *bla*$_{CTX-M-55}$, *fosA3*, *tetR*	AMX, CHL, CIP, CS, CTX, FOS, SXT, TET
CHD25*	ABD	453	*bla*$_{TEM-1B}$	AMP
CHD15	AxB1	162	*bla*$_{TEM-1B}$	AMP
CHD29*		399		
S115[#]	B1	23	*mcr-1*, *bla*$_{VIM-1}$, *bla*$_{CMY-2}$, *floR*	CIP, CHL, CS, IPM, SXT, IPM,
Af45[#]		101	*mcr-1*	CS, SXT, TET
CDF6[#]		446	*mcr-1*, *bla*$_{CTX-M-55}$, *fosA3*, *tetR*	AMX, CIP, CS, CTX, SXT, TET
CHD18*	B2	12		
ATCC 25922[#]*		73		
CHD3[#]*		73		
CHD27[#]*		73	*bla*$_{TEM-1B}$	AMP
83972[#]*		73		
UTI89[#]*		95		
CHD10		126		
CHD14*		127		
CHD2[#]		131	*bla*$_{TEM-1B}$	AMP
CHD5[#]*		131		
CHD16[#]		131	*bla*$_{TEM-1B}$, *bla*$_{CTX-M-15}$	AMP, CTX, GEN
CHD94[#]*		131		
CDF2[#]*		131	*mcr-1*, *bla*$_{TEM-1}$, *bla*$_{TEM-52}$	AMX, CS, CTX, GEN, NAL, SXT TET
H75[#]*		131	*bla*$_{CTX-M-15}$	AMP, CTX,
CHD13*		135		
CHD6*		141		
CHD8		5640	*bla*$_{TEM-1B}$	AMP
CHD17[#]	D	38	*bla*$_{CTX-M-15}$, *bla*$_{OXA-1}$	AMP, CTX, GEN
CHD11[#]		69		
CHD22[#]		69		
CHD23[#]		69		
CHD9		405	*bla*$_{TEM-1B}$, *bla*$_{CTX-M-14}$	AMP, CTX
Af31[#]*		624	*mcr-1*, *florR*	AMX, CHL, CIP, CS, SXT, TET
Af48[#]		624	*mcr-1*, *bla*$_{CMY-2}$	AMX, CEF, CIP, CS, KAN, SXT, TET
CHD20	D/F	648	*bla*$_{CTX-M-15}$	AMP, CTX
CHD21	F	117		
Af24	unknown	1007	*mcr-1*, *bla*$_{TEM-1}$	AMX, CHL, CIP, CS, SXT, TET
CHD4*		1064		
NRZ14408*		1851	*mcr-1*, *bla*$_{KPC}$	CIP, CS, IMP
CHD7*		2020	*bla*$_{TEM-1B}$	AMP
CDF1		3077	*mcr-1*, *bla*$_{TEM-1}$	AMX, CIP, CS, GEN, SXT, TET

IR3		not known	*bla*$_{NDM-1}$	AMP, CIP, CTX, IMP
MI-4*		not known	*nalR*	NAL
1949820		not known	*gyrA* S83N, *gyrA* D87N, *parC* S80I	CIP
523*		not known	*nalR*	NAL

# - strains used for breeding,* - strains used for efficiency determination of bred phages compared to ancestors, AMP – Ampicillin, AMX – Amoxicllin, CIP – ciprofloxacin, CHL, Chloramphenicol, CS – Colistin, CTX – Cefotaxim, FOS – Fosfomycin, GEN – Gentamicin, IMP – Imipenem, KAN – Kanamycin, NAL – Nalidixin acid, SXT – Trimethoprim-sulphamethoxazole, TET - tetracycline
