# Supplementary figures and images for: Natural Bred ε2-Phages Have an Improved Host Range and Virulence against Uropathogenic Escherichia coli over Their Ancestor Phages
Source: Antibiotics (Basel). 2021 Nov 1;10(11):1337. doi: 10.3390/antibiotics10111337 (PMC8614997; doi:10.3390/antibiotics10111337)

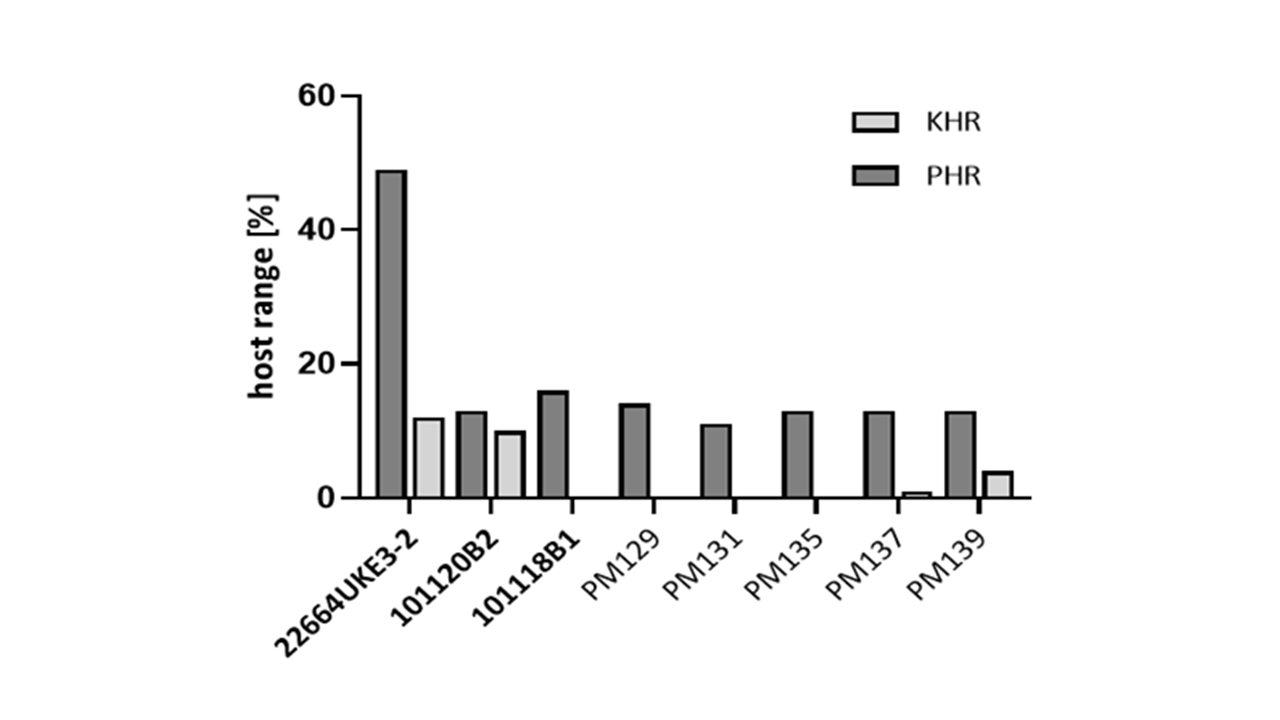

Supplement: Supplementary file 1 [file antibiotics-10-01337-s001.zip › Figure S1.tif]

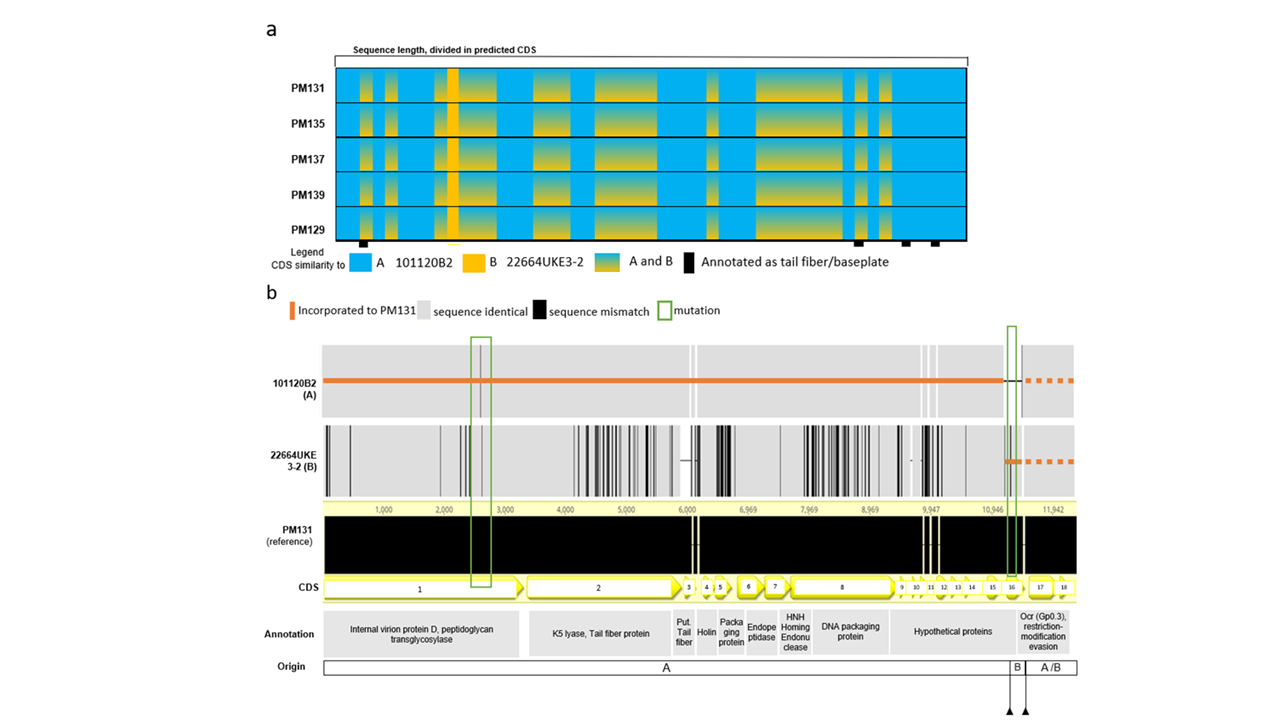

Supplement: Supplementary file 1 [file antibiotics-10-01337-s001.zip › Figure S2.tif]
